# The longitudinal interplay between negative and positive symptom trajectories in patients under antipsychotic treatment: a post hoc analysis of data from a randomized, 1-year pragmatic trial

**DOI:** 10.1186/1471-244X-13-320

**Published:** 2013-11-28

**Authors:** Lei Chen, Joseph A Johnston, Bruce J Kinon, Virginia Stauffer, Paul Succop, Tiago R Marques, Haya Ascher-Svanum

**Affiliations:** 1 Lilly Research Laboratories, Eli Lilly and Company, Lilly Corporate Center, Indianapolis, IN 46285, USA; 2 University of Cincinnati, Cincinnati, OH, USA; 3 Department of Psychosis Studies, Institute of Psychiatry, King’s College London, London, UK

**Keywords:** Positive symptoms, Negative symptoms, Trajectory interplay, Schizophrenia

## Abstract

**Background:**

Schizophrenia is a highly heterogeneous disorder with positive and negative symptoms being characteristic manifestations of the disease. While these two symptom domains are usually construed as distinct and orthogonal, little is known about the longitudinal pattern of negative symptoms and their linkage with the positive symptoms. This study assessed the temporal interplay between these two symptom domains and evaluated whether the improvements in these symptoms were inversely correlated or independent with each other.

**Methods:**

This post hoc analysis used data from a multicenter, randomized, open-label, 1-year pragmatic trial of patients with schizophrenia spectrum disorder who were treated with first- and second-generation antipsychotics in the usual clinical settings. Data from all treatment groups were pooled resulting in 399 patients with complete data on both the negative and positive subscale scores from the Positive and Negative Syndrome Scale (PANSS). Individual-based growth mixture modeling combined with interplay matrix was used to identify the latent trajectory patterns in terms of both the negative and positive symptoms. Pearson correlation coefficients were calculated to examine the relationship between the changes of these two symptom domains within each combined trajectory pattern.

**Results:**

We identified four distinct negative symptom trajectories and three positive symptom trajectories. The trajectory matrix formed 11 combined trajectory patterns, which evidenced that negative and positive symptom trajectories moved generally in parallel. Correlation coefficients for changes in negative and positive symptom subscale scores were positive and statistically significant (P < 0.05). Overall, the combined trajectories indicated three major distinct patterns: 1) dramatic and sustained early improvement in both negative and positive symptoms (n = 70, 18%), 2) mild and sustained improvement in negative and positive symptoms (n = 237, 59%), and 3) no improvement in either negative or positive symptoms (n = 82, 21%).

**Conclusions:**

This study of symptom trajectories over 1 year shows that changes in negative and positive symptoms were neither inversely nor independently related with each other. The positive association between these two symptom domains supports the notion that different symptom domains in schizophrenia may depend on each other through a unified upstream pathological disease process.

## Background

It has been long recognized that schizophrenia is a highly heterogeneous disease in terms of etiology, symptom manifestation, and treatment response [1-3]. Data from both randomized controlled trials [4-7] and a 30-year long observational study [8] has shown that treatment response is heterogeneous and is typically captured by four or five trajectory groups with symptom severity defined by the aggregated symptom scores. While the core symptoms of schizophrenia are characterized by positive (e.g., delusion, hallucination) and negative (e.g., emotional withdrawal) symptoms [9], it has been believed that currently available antipsychotics work primarily on relieving positive symptoms, whereas negative symptoms are harder to treat [10-13]. The schizophrenia research community has tremendous interest in understanding the nature of negative symptoms [8,14,15].

The literature, though, is limited regarding the longitudinal patterns of negative symptoms and their linkage with the positive symptoms. Previous studies on the relationship between negative and positive symptoms have been mainly conceptually driven or cross-sectional in nature [16]. It has been hypothesized that positive symptoms are related to an increase in dopamine D2 activity, while blockade of the dopamine D2 receptors may worsen negative symptoms, thus negative and positive symptoms might be inversely correlated [17-19]. It was also hypothesized that these two domains of symptomatology correspond to different etiopathogenic and pathophysiological mechanisms [20,21]. Concurrently, cross-sectional phenomenological studies have shown that there was no significant relationship between these two symptom domains, thus supporting the notion that these two symptom domains may be orthogonal or independent with each other [16,22]. On the other hand, hypotheses on the glutamatergic system suggest that glutamate is involved in the mediation of both positive and negative symptoms of schizophrenia in the same direction, although the data are inferential regarding drugs are currently in late stage clinical development [23].

In this study, we utilized an individual-based trajectory analysis technique to characterize the trajectories of negative and positive symptoms and assessed the temporal interplay between these two symptom domains by using data from a 1-year schizophrenia trial [24]. We seek to provide insight into the dynamic, individual-level interaction between negative and positive symptoms. Insight gained from these analyses could aid in management of patients with schizophrenia by raising awareness of the dynamic interplay between these two characteristic symptom domains and may help shed light on the pathological disease process of schizophrenia.

## Methods

### Study population

This analysis was based on data from a multicenter, randomized, open-label, 1-year pragmatic trial studying the cost-effectiveness of olanzapine as the first-line treatment of schizophrenia in the United States. The method and main results of this study have been published [24].

Study participants were recruited between 1998 and 2001 from both academic and community treatment settings, primarily in mental health outpatient clinics. Eligible patients were male and female, 18 years of age or older, met Diagnostic and Statistical Manual of Mental Disorders, Fourth Edition (DSM-IV) criteria for schizophrenia, schizoaffective disorder, or schizophreniform disorder and met criteria for psychotic-symptom exacerbation measured as a total Brief Psychiatric Rating Scale score of at least 18; or had recently experienced an adverse event (within 4 weeks with depo neuroleptic therapy or within 6 days with oral neuroleptic therapy) that was attributable to current antipsychotic treatment and who are no longer tolerating treatment. Patients with very serious, unstable physical illness and other medical conditions or histories contraindicating use of study drugs were excluded. Patients were randomized to olanzapine, risperidone, or first-generation antipsychotics (FGAs) at a 1:1:1 ratio. The study was designed to represent the usual clinical setting. Choice of the FGAs, initial dosing, titration, and dosing adjustments were determined by the treating physicians. Switching antipsychotic agents was also allowed and was at the discretion of the treating physician. Patients were assessed at seven visits, which corresponded to weeks 0, 1, 3, 9, 21, 33, and 49. The study protocol was approved by a central institutional review board (IRB; Western IRB, 3535 7th Ave SW, Olympia, WA 98502; Pr#: 980024) or by local IRBs, and patients signed consent before entering the study.

For the current post hoc study, the null hypothesis was that the improvement of negative and positive symptoms is either inversely related or independent in patients under antipsychotic treatment. Therefore, the analysis was conducted using samples with complete 1-year data on the Positive and Negative Syndrome Scale (PANSS) negative subscale (N = 400) and the positive subscale scores (N = 401), with an overall sample size of 399 patients with complete data on both subscales. Data from different medication groups were pooled. Table 1 lists the patient demographics, diagnosis, baseline illness characteristics, and symptom severity.

**Table 1:** Baseline patient characteristics

**Variable**	**Overall**^**a**^
Age (years), mean (SD)	43.8 (12.0)
Male, n (%)	244 (61.2%)
Race/ethnicity, n (%)	
Caucasian	236 (59.1%)
African American	118 (29.6%)
Other	45 (11.3%)
Currently employed, n (%)	84 (21.1%)
Inpatient setting at trial entry, n (%)	15 (3.8%)
Primary psychiatric diagnosis, n (%)	
Schizophrenia	265 (66.4%)
Schizophreniform	3 (0.8%)
Schizoaffective disorder	131 (32.8%)
Age at first psychiatric hospitalization (years), mean (SD)	26.4 (9.4)
Number of previous episodes of schizophrenia, mean (SD)	6.4 (8.6)
Antipsychotic treatment in the past year, n (%)	
Conventional(s) only	245 (61.4%)
Atypical(s) only	40 (10.0%)
Both	81 (20.3%)
Comorbid psychiatric diagnosis, n (%)	
Mood disorder	86 (21.6%)
Anxiety disorder	19 (4.8%)
Psychoactive substance use disorder	150 (37.7%)
PANSS total score, mean (SD)	86.2 (19.7)
BPRS total score, mean (SD)	31.4 (11.5)

*Abbreviations*: *BPRS* Brief Psychiatric Rating Scale, *PANSS* Positive and Negative Syndrome Scale, *SD* standard deviation.

^a^n = 399 for age, gender, race/ethnicity, primary psychiatric diagnosis, inpatient setting at trial entry, currently employed, antipsychotic treatment in the past year, PANSS, and BPRS; n = 362 for age at first psychiatric hospitalization; n = 392 for previous episodes of schizophrenia; n = 398 for comorbid psychiatric diagnoses.

### Measures

The primary outcome measures of negative and positive symptom severity levels were assessed at every study visit using the PANSS negative and positive subscale scores, as defined by Kay et al. [25]. The PANSS is a 30-item, clinician-rated instrument with each item scored in an incremental seven-point severity scale (from 1 = absent, 2 = minimal, to up to 7 = extreme). The positive subscale score is calculated as the sum of seven positive items, and the negative subscale score is the sum of the seven negative items. Thus, the negative and positive subscale scores each range from 7 to 49. The PANSS was developed in the 1980s as a well operationalized instrument that provides balanced representation of negative and positive symptoms [25,26]. The psychometric properties of PANSS had been studied and proved reliable and valid [26,27]. The PANSS has been widely used in the research of schizophrenia and is accepted by the Food and Drug Administration as the primary efficacy outcome measure for new drug applications treating schizophrenic spectrum disorder [28-30].

### Statistical analyses

We used growth mixture modeling (GMM) [31] to model PANSS negative and positive subscale scores. Growth mixture modeling is an individual-based modeling technique that permits investigators to explore the longitudinal features of disease progression (i.e., symptom trajectories) and to cluster patients into latent classes (subgroups) based on the differential symptom courses [32]. GMM allows the assumption that there exist a certain number of distinct pathways of growth or disease progression; therefore, subjects can be grouped into a small number of distinct clusters based on their disease progression profile [33]. The trajectory classes are not defined *a priori* but are inferred from the data; thus, the trajectory classes are also called latent classes. Mathematically, GMM employs both categorical and random-effect continuous latent variables to capture population heterogeneity in the disease progression. The categorical latent variables represent different trajectory patterns (or classes), while the class-varying random-effect continuous latent variables capture heterogeneity among individuals within the class. For our study, we first applied GMM on PANSS negative and positive subscale scores, separately, using a series of models that included random effects for intercept, linear slope, and quadratic slope. Multiple statistical criteria (i.e., Bayesian information criterion [BIC], sample-size-adjusted BIC [aBIC], and bootstrap likelihood ratio test [BLRT]) were used to determine the optimal number of latent trajectory classes.

Secondarily, we generated a matrix of PANSS negative symptom trajectories versus positive symptom trajectories to create patient groups based on the combined symptom trajectories (e.g., if a patient falls into Class 1 for negative symptom trajectories and Class 2 for positive symptom trajectories, then the patient would belong to Group 1–2 of the combined symptom trajectories).

To further examine the relationship between negative and positive symptoms, we calculated Pearson correlation coefficients [34] between the changes in the two subscale scores within each matrix cell by visit intervals after randomization.

## Results

### Negative symptom trajectories

**Figure 1:**
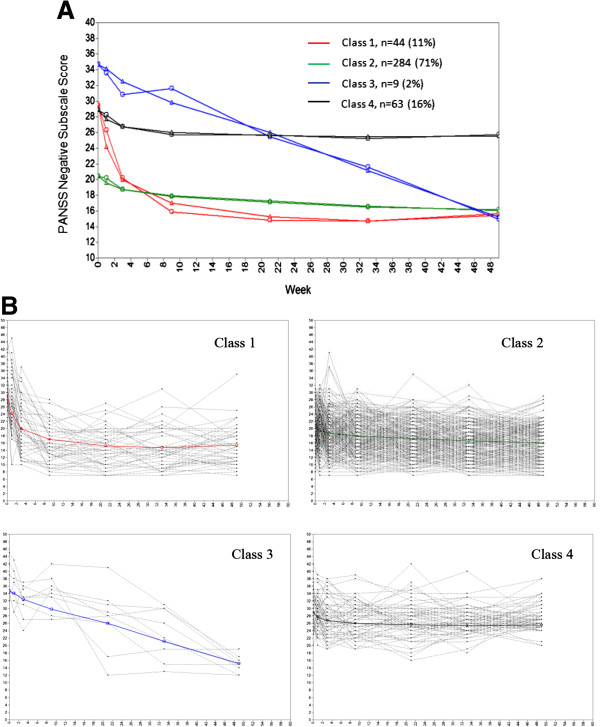
**Negative symptom trajectories. A**: Estimated and observed mean curves. Triangles indicate estimated means, and circles indicate observed means. **B**: Individual profiles by negative symptom trajectories. Light gray lines show trajectory of negative symptom subscale for each patient in each latent class. Bold lines show observed mean trajectories of the corresponding latent classes.

To identify the different trajectory subtypes in terms of negative symptoms, data from 400 patients with complete 1-year PANSS negative subscale scores were fit to a sequential series of quadratic growth models that reflected one to five different trajectory latent classes. The statistical indices associated with the series of models (i.e., one to five latent classes) are shown in Table 2. Per the aBIC (the lower, the better) and BLRT, the four-trajectory model outperformed the other models. Figure 1A shows the observed and estimated mean PANSS negative subscale scores by the latent classes of the four-trajectory solution. There were 44, 284, 9, and 63 patients in each latent class, which represented 11%, 71%, 2%, and 16% of the entire cohort, respectively. Although the smallest group accounts for only 2% of the patients, its symptom profile is exclusive (i.e., a continuous and robust response in PANSS negative subscale score through the course of the study). Thus, we chose to keep this distinct group and, as such, the four-trajectory solution. Figure 1B shows the trajectory of the negative symptom subscale for each patient in each latent class and the observed mean trajectory of the corresponding latent class. The mean trajectory of each class demonstrated a reasonable level of concordance with individual patient trajectories and provided straightforward evidence supporting the four-trajectory solution.

**Table 2:** The fit statistics for the different GMM sequential models explored for negative symptoms

**Number of classes**	**1**	**2**	**3**	**4**	**5**
BIC	16518	16517	16522	16526	16548
aBIC	16467	16453	16445	16437	16446
BLRT	N/A	<0.001	<0.001	<0.001	0.167
Number of patients in each class	400	378/22	22/374/4	44/284/9/63	16/3/56/319/6

*Abbreviations*: *GMM* growth mixture modeling, *BIC* Bayesian information criterion, *aBIC* sample-size-adjusted Bayesian information criterion, *BLRT* bootstrap likelihood ratio test, *N/A* not applicable.

### Positive symptom trajectories

**Figure 2:**
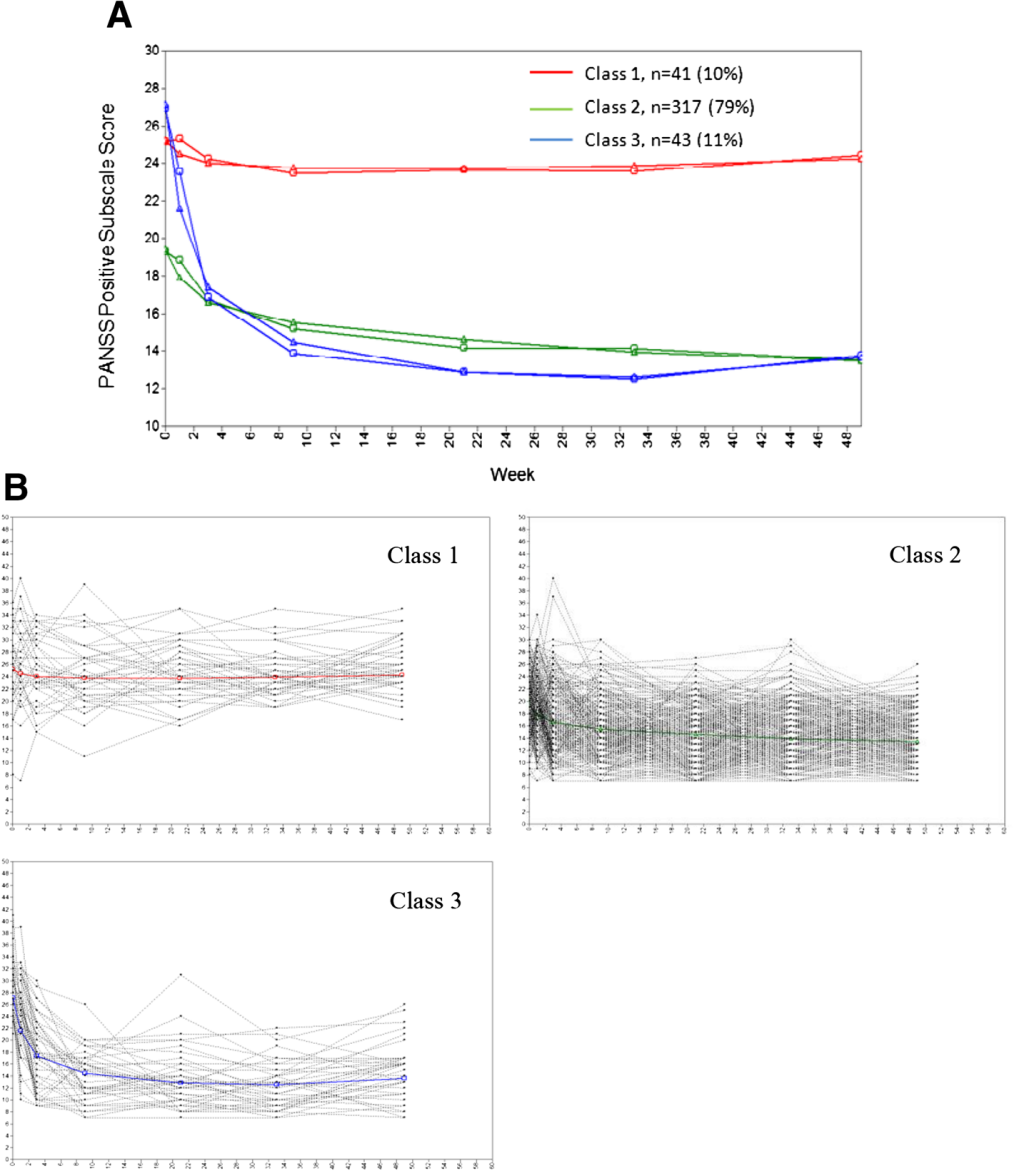
**Positive symptom trajectories. A**: Estimated and observed mean curves. Triangles indicate estimated means, and circles indicate observed means. **B**: Individual profiles by positive symptom trajectories. Light gray lines show trajectory of positive symptom subscale for each patient in each latent class. Bold lines show observed mean trajectories of the corresponding latent classes.

Likewise, we modeled a sequential series of quadratic growth models for the positive symptoms with 401 patients who have complete data on the PANSS positive subscale scores. The statistical indices associated with the series of models (i.e., one to four latent trajectories) are shown in Table 3. Per BIC, the three-trajectory model outperformed the others. Although the BLRT showed a significant difference of the four-class model versus the three-class model (P < 0.001), the fourth class only accounted for 1.5% (n = 6) of the patients and this fourth class did not evidence a distinct symptom profile; thus, the three-class model was chosen. With this three-class model, the class sizes were of reasonable magnitude for interpretation with 41 (10%), 317 (79%), and 43 (11%) of patients in each latent class. In addition, the three-class solution demonstrated a reasonable level of concordance between each class mean trajectory and individual patient trajectories within the class (Figure 2A and B).

**Table 3:** The fit statistics for the different GMM sequential models explored for positive symptoms

**Number of classes**	**1**	**2**	**3**	**4**
BIC	16091	16066	16064	16070
aBIC	16040	16002	15988	15981
BLRT	N/A	<0.001	<0.001	<0.001
Number of patients in each class	401	42/359	41/317/43	39/94/262/6

*Abbreviations*: *GMM* growth mixture modeling, *BIC* Bayesian information criterion, *aBIC* sample-size-adjusted Bayesian information criterion, *BLRT* bootstrap likelihood ratio test, *N/A* not applicable.

### Temporal interplay between negative and positive symptoms

**Figure 3:**
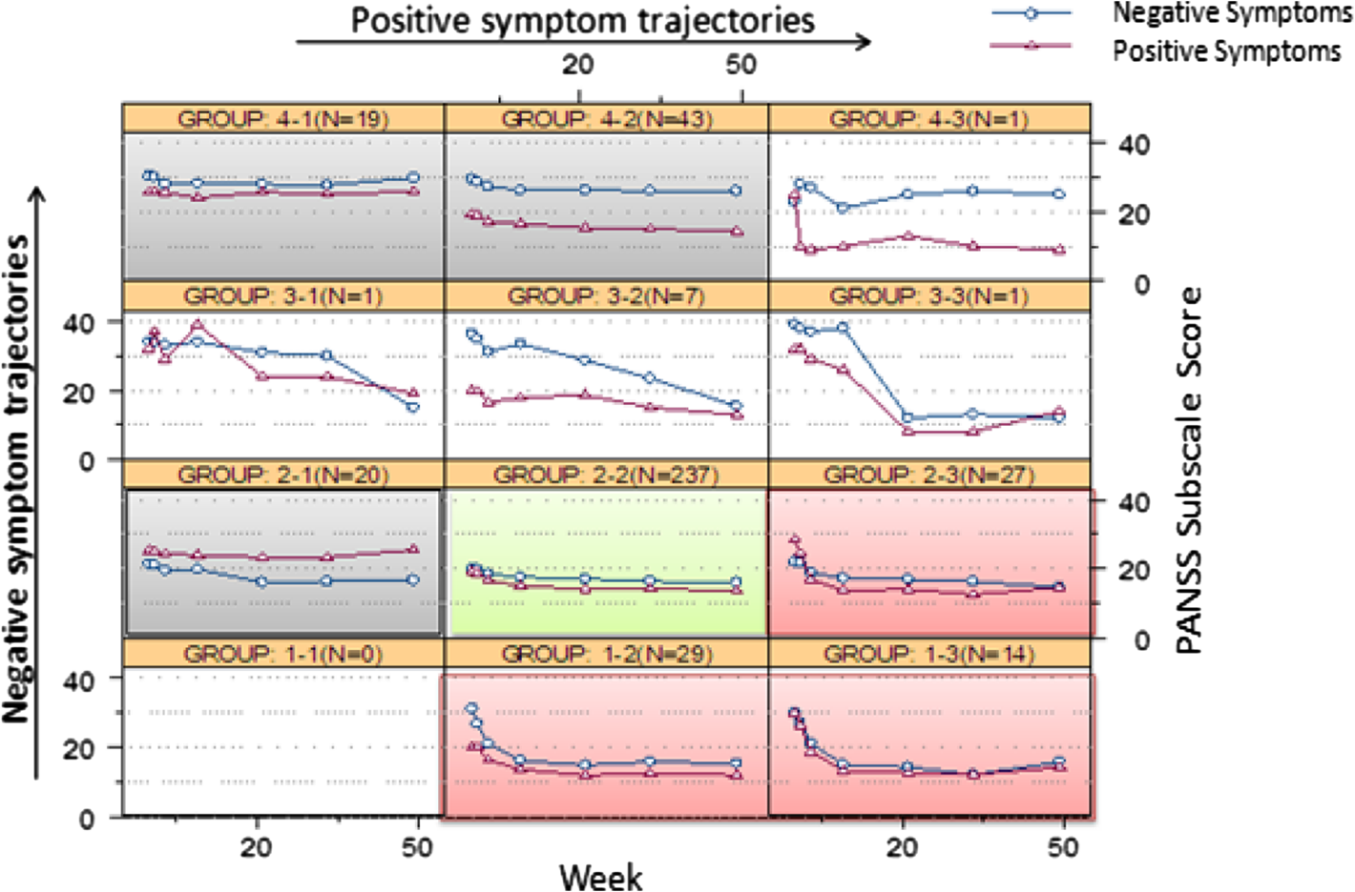
**Interplay matrix of negative and positive symptom trajectories.** Abbreviations: DSI = dramatic and sustained early improvement, MSI = mild and sustained improvement, NI = no improvement. The pink cells reflect DSI, the green cells reflect MSI, the gray cells reflect NI, and the uncolored cells reflect idiosyncratic trajectories.

Figure 3 shows the negative and positive symptom trajectory matrix. The four (negative symptom trajectory classes) by three (positive symptom trajectory classes) matrix formed 12 cells with one empty cell (cell 1–1, no patient fell into this category), three cells (cells 3–1, 3–3, and 4–3) with only one patient each, one cell (cell 3–2) with seven patients, and the remaining seven cells with at least 14 patients each.

The trajectory matrix indicates that, within each matrix cell (or patient groups), negative and positive symptom trajectories tend to move in parallel over time (except in two idiosyncratic individual cases of cells 3–1 and 4–3, which account for two patients in total, 0.5% of the studied population), while the negative symptom subscale scores tend to be consistently higher than positive symptom subscale scores (except cell 2–1, which accounts for 20 patients, 5% of the studied population).

Pearson correlation coefficients between change in PANSS negative and positive symptoms are shown in Table 4 by visit interval within each of the seven matrix cells, which contained at least 14 patients each. Positive relationships were observed with 33 of the 35 studied matrix cell by visit interval combinations. While the two negative correlations (cell 4–2 at visit intervals week 9–21 and week 21–33) were numerically small and statistically non-significant, statistically significant, moderate to large correlations were evidenced at some visit intervals for each of the studied matrix cells.

**Table 4:** Pearson correlation coefficients between change in PANSS negative and positive symptom subscale scores by interplay matrix groups

**Visit interval**	**Group 1–2**	**Group 1–3**	**Group 2–1**	**Group 2–2**	**Group 2–3**	**Group 4–1**	**Group 4–2**
	**(N = 29)**	**(N = 14)**	**(N = 20)**	**(N = 237)**	**(N = 27)**	**(N = 19)**	**(N = 43)**
Week 1-3	0.65^a^	0.59^a^	0.60^a^	0.30^a^	0.02	0.41	0.58^a^
Week 3-9	0.36	0.61^a^	0.20	0.39^a^	0.39^a^	0.58^a^	0.35^a^
Week 9-21	0.69^a^	0.63^a^	0.32	0.22^a^	0.11	0.62^a^	-0.08
Week 21-33	0.56^a^	0.47	0.42	0.31^a^	0.54^a^	0.59^a^	-0.19
Week 33-49	0.23	0.61^a^	0.42	0.36^a^	0.34	0.35	0.05

^a^P < 0.05.

Overall, the combined negative and positive symptom trajectories suggested that 98% of the population generally experienced one of three distinct patterns: 1) dramatic and sustained early improvement (DSI) in both negative and positive symptoms (cells 1–2, 1–3, and 2–3; n = 70, 18%); 2) mild and sustained improvement (MSI) in negative and positive symptoms (cell 2–2; n = 237, 59%); or 3) no improvement (NI) in either negative or positive symptoms (cells 2–1, 4–1, and 4–2; n = 82, 21%). Two percent of the patients followed idiosyncratic courses (cells 3–1, 3–2, 3–3 and 4–3, n = 10).

## Discussion

This study employed an individual-based trajectory analysis method to study the temporal relationship between negative and positive symptoms. We observed that negative and positive symptom trajectories tended to move in parallel over the 1-year study for most of the 11 patterns of combined latent trajectory classes, and correlation between the changes of PANSS negative and positive symptoms was significant as demonstrated by Pearson correlation coefficients. These findings suggest that changes in negative and positive symptoms are neither inversely related nor independent with each other, at least in chronically ill patients treated in the United States, who represent the study population.

To the authors’ knowledge, the present study is the first to assess the differential symptom courses simultaneously using both negative and positive symptoms over 1 year of antipsychotic treatment. The pattern (latent trajectory classes) was mathematically derived using an algorithm designed to group patients into clusters so that the symptomatology pattern over 1 year is relatively homogeneous in each class. There was no preconceived notion (e.g., baseline characteristics) used to assign patients to one trajectory versus another. The adoption of the trajectory analysis makes possible, for the first time, the analyses and inspection of the relationship between these two symptom domains taking into account both the dynamic and heterogeneous nature of the symptom manifestation [35,36]. In our previous research, we applied trajectory analysis to schizophrenia clinical trial data and identified subgroups of patients with relatively homogeneous treatment-response patterns in terms of the PANSS total score or positive symptom subscale score [3,36,37], but this technique had yet to be applied on negative and positive symptoms simultaneously.

The congruence observed for longitudinal change in negative and positive symptoms was not anticipated per theories that these two sets of symptom domains might be reversely correlated [19] or be independent with each other [20,38]. Although the underlying mechanism of this phenomenon is not well understood, our finding might be explained by the newly reconceptualized dopamine hypothesis proposed by Howes and Kapur [39]. Based on the emerging evidence in imaging, genetic, environmental, phenotypic, and animal studies in the past two decades, Howes and Kapur hypothesized that various symptoms in schizophrenia are systematical manifestations of the downstream neurotransmitter abnormality (i.e., the dopamine dysregulation), and different symptom domains may depend on each other through a unified upstream pathological disease process [39]. Our findings that changes n negative and positive symptoms were positively related may be a reflection of a unified pathological disease process.

Furthermore, our findings corroborate those reported by Addington and Addington [16] who studied 41 patients with DSM III-diagnosed schizophrenia and assessed the relationship between negative and positive symptoms measured by the Scale for the Assessment of Negative Symptoms and the Scale for the Assessment of the Positive Symptoms. Their study found positive correlations between these two symptom domains at both the acute phase and remission phase and concluded that negative and positive symptoms are not inversely related.

We observed that negative and positive symptoms moved in parallel over the 1-year period for most of the 11 patterns of combined latent trajectory classes. It could be argued that the congruence of these two symptom domains may be confounded by measurement error at the level of rating (i.e., a high score on one dimension leads to an erroneous high rating on another dimension; therefore, as the score on one dimension improves the score on the other dimension also seems to improve). While the possibility of measurement error cannot be ruled out in the context of an observational study, we think it is rather unlikely in our study as the correlation between the two dimensions was seen across time, and the trajectory subgroups have been observed to be directionally consistent with 1-year functional and health-related quality of life outcomes in a follow-up analysis (data not shown).

It may also be argued that the observed congruence between negative and positive symptom trajectories may be a reflection of the secondary negative symptoms, the effect on negative symptoms that associate with positive symptoms, extrapyramidal side effects, or mood [40]. Again, we think the concept of secondary negative symptoms is not likely to explain all the congruence between these two symptom trajectories observed in our study. Tollefson and Sanger [41] conducted a path analysis to tease out the secondary effect of positive symptoms, mood, or adverse events and found that negative symptoms of schizophrenia are directly responsive to treatment. Stauffer et al. [42] studied primary negative symptoms using the proxy of predominant negative symptoms precluding the effect of positive symptoms, depressive symptoms, and parkinsonism, but the study showed that such segregation of patients does not suggest prognostic implications.

From a clinical perspective, negative symptoms, regardless of being primary or secondary in nature, as a whole are an indication of disease severity [43] and are significantly related to quality of life and level of functioning [44,45]. Our findings agree with findings from a majority of the studies on treatment with second-generation antipsychotics in which both negative and positive symptoms improved at the population level [28-30,46-48]. Further, our findings suggest that, even at the individual patient level, improvements in both negative and positive symptoms are achievable, although there is still a considerable unmet medical need in term of the magnitude of the improvement in both the negative and positive symptoms. The perception that negative symptoms do not improve compared to positive symptoms may not be totally accurate.

Moreover, we observed that negative symptom subscale scores tended to be consistently higher than positive symptom subscale scores. Although the clinical meaning of a certain score on the PANSS negative or positive subscale is not clear, this observation is consistent with the previous findings that populations with chronic schizophrenia have higher negative symptoms [45,49] and if they improve in concert, it is understandable that their severity will continue to be higher. Exceptionally, one small group of 20 patients (5% of the overall studied population) showed higher positive than negative symptom subscale scores. It would be interesting to understand the characteristics of such a group of patients in a larger database.

Lastly, the data suggests that the majority of patients generally experience one of the three distinct trajectory patterns of treatment-response course: 1) dramatic and sustained early improvement in both negative and positive symptoms, 2) mild and sustained improvement in negative and positive symptoms, or 3) no improvement in either negative or positive symptoms. Further research on the association between these treatment response trajectory subgroups and the underlying determinants (i.e., treatment choice, pathophysiology, and etiology indicators) may help inform clinical practice or aid the effort to develop targeted treatment of schizophrenia.

This study has a number of pertinent strengths and limitations that warrant discussion. First, this study is novel; the association between the two symptom domains evidenced at the individual patient level and these analyses have not been executed by others in this fashion. The field has been accustomed to seeing data at the population level (i.e., treatment group), an approach that does not necessarily imply that negative and positive symptom response is congruent. This study shows, however, at the individual patient level, the two symptom domains in fact do appear to display a commonality of response. Second, results from this study would apply in real-world practice settings. The data source is an open-label, 1-year, pragmatic trial. The study sample included a heterogeneous patient population with a variety of comorbid conditions, such as substance abuse; physicians were allowed to adjust dosage or switch medications according to their clinical discretion. Thus, the data reflect usual clinical care.

On the other hand, the study is limited as a post hoc, exploratory study. First, this study included patients who were mostly chronically ill; therefore, the findings may not be generalizable to patients in their early stage of the illness. Second, the treatment was open-label and drug switching was allowed. As such, we did not extend this study to analysis and inference on the specific drug effect, which is a clinically important question. Thirdly, we recognize that the definition and construct of negative and positive symptoms remains a subject of debate, though PANSS negative and positive subscale scores have been extensively used in schizophrenia clinical trials. Lastly, this study was not able to differentiate primary versus secondary negative symptoms due to the operational complexity of the concept. We expect future studies employing an advanced analytical method would help shed light on this issue. Overall, replication of the finding using independent data is necessary.

## Conclusions

In summary, this study found that negative and positive symptom trajectories move in parallel over time, and changes in these two symptom domains are positively correlated in patients undergoing antipsychotic treatment. These findings support the Howes-Kapur hypothesis that there may be a common upstream pathological process that leads to different symptom manifestations in schizophrenia.

## Competing interests

LC, JAJ, BJK, VS, and HA are employees and current stakeholders of Eli Lilly and Company. PS is a faculty member at the University of Cincinnati. TRM is employee of King’s College London. PS and TRM were not paid for their intellectual contributions to the study and have no competing interest to report.

## Authors’ contributions

LC conceived the study. LC and JAJ contributed to the initial design and coordination. LC led the statistical analysis, wrote the initial draft of the manuscript, and coordinated the development of subsequent drafts. All authors participated in the analysis and interpretation of the data, as well as revising the manuscript for critically important intellectual content. In addition, all authors read and approved the final version of the manuscript.

## Pre-publication history

The pre-publication history for this paper can be accessed here:

http://www.biomedcentral.com/1471-244X/13/320/prepub

## Local IRBs approved study F1D-US-HGGD

St. Davids Human Research Review Board, One Corporate Center Suite 300, Radnor PA 19087; IRB for Human Subjects Research for Baylor College, One Baylor Plaza, Houston TX 77030; McLean IRB- McLean Hospital, 115 Mill Street, Belmont, MA 02478; UMKC Adult Health Sciences IRB, Truman Medical Center 2301 Holmes, Kansas City MO 64104. Street St.Paul, MN 55101; State of Utah, 120 North 200 West #319 Salt Lake City, Utah 84103.
